# Three-year follow-up analysis of the short-stitch versus long-stitch technique for elective midline abdominal closure randomized-controlled (ESTOIH) trial

**DOI:** 10.1007/s10029-024-03025-9

**Published:** 2024-03-27

**Authors:** R. H. Fortelny, A. Hofmann, P. Baumann, S. Riedl, J. L. Kewer, J. Hoelderle, A. Shamiyeh, B. Klugsberger, T. D. Maier, G. Schumacher, F. Köckerling, Ursula Pession, M. Schirren, M. Albertsmeier

**Affiliations:** 1 Allgemein-, Viszeral- und Tumorchirurgie, Wilhelminenspital Montleartstr. 37, 1160 Vienna, Austria; 2 Department of General, Visceral and Transplantation Surgery, Ludwig-Maximilians-Universität (LMU) Munich, LMU University Hospital, 81377 Munich, Germany; 3 Department of Medical Scientific Affairs, Aesculap AG, Am Aesculap Platz, 78532 Tuttlingen, Germany; 4 Alb Fils Klinik GmbH, Klinik am Eichert, Allgemeinchirurgie, Eichertstr. 3, 73035 Göppingen, Germany; 5 Klinikum Landkreis Tuttlingen, Viszeral- und Gefäßchirurgie, Klinik für Allgemein, Zeppelinstr. 21, 78532 Tuttlingen, Germany; 6 Kepler Universitätsklinikum GmbH, Klinik für Allgemein- und Viszeralchirurgie, Krankenhausstr. 9, 4021 Linz, Austria; 7 Robert-Bosch-Krankenhaus, Allgemein- und Viszeralchirurgie, Auerbachstr. 110, 70376 Stuttgart, Germany; 8 Städtisches Klinikum Braunschweig, Chirurgische Klinik, Salzdahlumer Str. 90, 38126 Brunswick, Germany; 9 Vivantes Humboldt-Hospital, Hernia Center, Am Nordgraben 2, 13509 Berlin, Germany; 10 Zentrum der Chirurgie, Klinik für Allgemein- und Viszeralchirurgie, Universitätsklinikum Frankfurt, Theodor-Stern-Kai, 60590 Frankfurt am Main, Germany; 11 Med. Fakultät, Sigmund Freud Privatuniversität, Freudplatz 3, 1020 Vienna, Austria

**Keywords:** Randomised-controlled trial, Prevention, Incisional hernia, Laparotomy, Short stitches, Small bites

## Abstract

**Background:**

Clinical trials have shown reduced incisional hernia rates 1 year after elective median laparotomy closure using a short-stitch technique. With hernia development continuing beyond the first postoperative year, we aimed to compare incisional hernias 3 years after midline closure using short or long stitches in patients from the ESTOIH trial.

**Methods:**

The ESTOIH trial was a prospective, multicenter, parallel-group, double-blind, randomized-controlled study of primary elective midline closure. Patients were randomized to fascia closure using a short- or long-stitch technique with a poly-4-hydroxybutyrate-based suture. A predefined 3-year follow-up analysis was performed with the radiological imaging-verified incisional hernia rate as the primary endpoint.

**Results:**

The 3-year intention-to-treat follow-up cohort consisted of 414 patients (210 short-stitch and 204 long-stitch technique) for analysis. Compared with 1 year postoperatively, incisional hernias increased from 4.83% (20/414 patients) to 9.02% (36/399 patients, *p* = 0.0183). The difference between the treatment groups at 3 years (short vs. long stitches, 15/198 patients (7.58%) vs. 21/201 (10.45%)) was not significant (OR, 1.4233; 95% CI [0.7112–2.8485]; *p* = 0.31).

**Conclusion:**

Hernia rates increased significantly between one and 3 years postoperatively. The short-stitch technique using a poly-4-hydroxybutyrate-based suture is safe in the long term, while no significant advantage was found at 3 years postoperatively compared with the standard long-stitch technique.

**Trial registry:**

NCT01965249, registered on 18 October 2013.

## Introduction

During their lifetime, one-third of the population in industrialized countries will undergo abdominal surgery [1]. For those above 60 years of age, this rate increases to over 43%. Ventral incisional hernias cause considerable morbidity in the affected patients. An increase in the size of hernias often results in a deterioration of functional capacities, quality of life, and body image [2]. Furthermore, some patients develop skin defects, which are difficult to treat. Finally, the most severe complication is entrapment and strangulation of the contents of the hernial sac, which requires emergency surgery. It is well known that the short- and long-term results of such emergency procedures are significantly worse than those of elective hernia surgery, which per se can be associated with wound healing disorders and (recurrent) incisional hernia rates of 20–30% in each case [3].

Despite the general trend toward minimally invasive laparoscopic surgery, midline laparotomy remains the most frequent approach to the abdominal cavity, especially in oncological visceral, gynecologic, and urologic surgeries. Open repair of the abdominal aorta, which is primarily indicated for acute cases, is another procedure performed via the linea alba. In addition to burst abdomen in the early postoperative period, incisional hernias are the main complications.

Their incidence after midline laparotomy is reported to be 11–20% at a mean follow-up of 12 and 20 months, respectively [4]. However, with a longer follow-up, more incisional hernias are detected. However, two studies found a relative increase in hernia incidence of > 60% between 1 and 3 years (13.1–21.3%) [5]. It is estimated that, referring to the final percentage of incisional hernias in long-term follow-up, approximately 55% can be detected within 1 year, 75% within 2 years, and only within 5 years about 90% of incisional hernias will have occurred [4, 6]. Together, these data imply that the incidence of incisional hernia has been significantly underestimated in previous trials with a short 1-year follow-up and that a longer follow-up is required [5]. Although it is unlikely that a change in the hernia rate ratio between short- and long-term techniques will occur, valid estimates of actual hernia rates are a decisive factor for the level of evidence attributed to clinical data.

## Methods

The study design, participant inclusion and exclusion criteria, intervention details, randomization, and the statistics of the Effects of Short-Stitch Technique on the Occurrence of Incisional Hernia (ESTOIH) trial were described in the published study protocol [7] and previous reports [8, 9].

### Trial design

The ESTOIH trial was designed as a multicenter, double-blind, controlled study with 1:1 randomization in Germany and Austria. This study was registered at ClinicalTrials.gov on 13 October 2013 (NCT01965249). This study was approved by all institutional review boards at the participating centers and was conducted according to the ethical standards outlined in the 1964 Declaration of Helsinki and its subsequent amendments.

### Participants

The study included patients aged 18 years or older, with an American Society of Anesthesiologists score of I–III, scheduled for visceral surgery through primary midline laparotomy with an incision length of ≥ 15 cm and an expected 1-year survival. The exclusion criteria were emergency surgery, BMI ≥ 30 kg/m^2^, pancreatic tumors, abdominal aortic aneurysm surgery, and conditions such as peritonitis, coagulopathy, immunosuppressive therapy (> 40 mg corticosteroid or azathioprine), chemotherapy within 2 weeks before surgery, and abdominal radiation therapy within 8 weeks before surgery. Pregnant women, patients with severe neurologic/psychiatric comorbidities, and those with poor compliance were excluded.

All participants provided written informed consent. When recruitment fell short, two protocol changes were implemented: the BMI exclusion criterion was removed for cohort homogeneity, and the exclusion criterion “pancreatic tumor patients” became “pancreatic cancer patients” to allow benign tumor inclusion.

Patients were recruited from nine study centers in Germany and Austria, including three university hospitals, three tertiary referral centers, and three local/regional hospitals.

### Interventions

After incision of the skin and subcutis, subcutaneous adipose tissue removal extended at least 1 cm from the linea alba in all directions. The umbilical stalk was routinely dissected from the aponeurosis and re-fixed after fascial closure. For closure of the rectus fascia, an elastic, extra-long-lasting, absorbable, monofilament suture material made of poly-4-hydroxybutyrate (MonoMax^®^) was used in both study groups.

In the long-stitch group, MonoMax^®^ USP 1 loops with an HR 48 mm needle were used, employing a continuous suture technique with 10 mm stitch intervals and a 10 mm distance from the wound edge. The suture length-to-wound length ratio (SL:WL ratio) was 4:1, with overlapping and separate knots. The short-stitch group used MonoMax^®^ USP 2/0 with an HR 26 mm needle, applying a single continuous suture with 5 mm stitch spacing and 5–8 mm distance from the wound edge, achieving an SL:WL ratio of at least 5:1.

Surgeons were trained on-site by the principal investigator (R.H.F.) before the study, and additional training videos were provided to all trial sites. A study nurse recorded the stitch count and suture time. Suture technique parameters (SL:WL ratio) were recorded in case reports and monitored during site visits, with deviations addressed in study group meetings to ensure homogeneity.

### Outcome measures

#### Primary outcome

The primary outcome of the ESTOIH trial was the incisional hernia rate after 1 year. Follow-up analyses were planned after three and 5 years. The definition of incisional hernia was analogous to the European Hernia Society (EHS) as “abdominal wall hernia with or without protrusion in the area of the postoperative scar that is perceptible or palpable by clinical examination or imaging” [10]. To guarantee the quality and safety of the follow-up examination regarding the exclusion or detection of an incisional hernia, an ultrasound examination of the abdominal wall was performed in addition to a clinical examination. If a patient underwent routine cross-sectional imaging (CT or MRI) during follow-up, no additional ultrasound examination was performed.

#### Secondary outcomes

Quality of life was analyzed using the EQ-5D-5L questionnaire [11] preoperatively and at 30 days, 1 year, and 3 years postoperatively. Short-term complications such as surgical site infections (SSI), ruptured abdominal wounds, wound healing disorders, seromas, hematomas, and other adverse events not directly related to wound healing, as well as the length of hospital stay, have been reported previously [8].

### Sample size calculation

The ESTOIH study’s sample size determination was based on the ISSAAC study [12], which revealed a 19% 1-year incisional hernia risk using the long-stitch technique. The primary objective was to demonstrate a 50% reduction in the 1-year incisional hernia rate with the short-stitch technique compared to that with the long-stitch technique. Assuming an absolute risk decrease from 19 to 9.5%, a power of 80%, and an alpha error of 5%, the sample size was calculated to be 424 patients (212 per group), with a planned enrollment of 468, considering a 10% dropout rate. There were no replacements for dropped-out patients. Recruitment was capped at 200 patients per center to mitigate center effects. Recruitment was concluded after an interim analysis of the primary outcome when 424 patients were randomized according to the initial sample size calculation without substituting for early terminations.

### Randomization and blinding

Patients were randomized intraoperatively just before abdominal wall closure using sealed envelopes provided by the sponsor, achieving a 1:1 allocation ratio to short- or long-stitch suture techniques. The randomization lists, generated for each study center with varying block lengths using SAS 9.1, a statistical software (SAS Institute Inc., Cary, NC, USA), were securely sealed and stored at trial sites. Both patients and outcome assessors were blinded to the group allocation. The observer, who lacked access to the randomization list, received case report forms from a person (e.g., a study nurse) not involved in the outcome assessment. Surgeons performing abdominal wall closures were unblinded but not part of the outcome assessment process.

## Results

### Patients

**Fig. 1:**
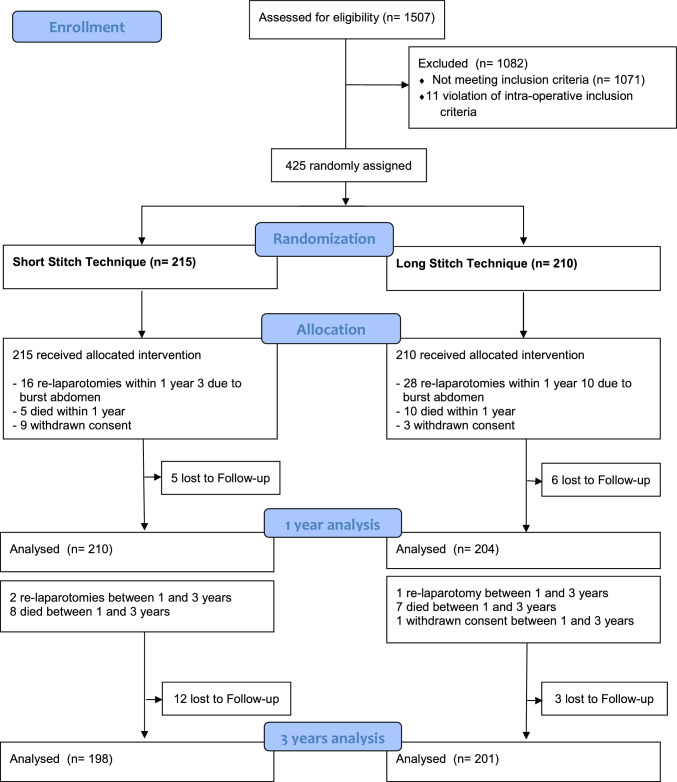
CONSORT flow chart ESTOIH 3 years

Between March 2014 and December 2019, 425 patients were randomized: 215 patients to the short-stitch technique and 210 to the long-stitch techniques, respectively. The study groups were similar in terms of baseline clinical data and procedural characteristics. Demographic data of the ESTOIH study population have been previously reported [8]. Short-term complications, including surgical site infections and burst abdomen until 30 days after surgery, have been published previously [8], as well as the primary outcome of incisional hernia 1 year postoperatively [9]. In total, 414 patients were analyzed in the intention-to-treat analysis and 323 patients in the per-protocol analysis 1 year postoperatively (Table 1). Three years after surgery, the number of patients under investigation had decreased to 399 in the intention-to-treat analysis (short-stitch group, *N* = 198 patients vs. long-stitch group, *N* = 201 patients). The CONSORT flow chart is depicted in Fig. 1. The per-protocol analysis comprised 273 patients available for examination: 139 in the short-stitch group and 134 in the long-stitch group. From surgery until 3 years postoperatively, a total of 76 subjects were not available for the 3-year examination in the short-stitch group for the following reasons: 17 patients were lost to FU, 13 died, nine withdrew, 18 patients received a relaparotomy, and 19 cases prematurely completed the study for reasons other than the long-stitch group: 9 lost to FU, 17 deaths, four withdrawals, 29 relaparotomies, and 17 other reasons.

**Table 1:** Hernia rates 1-year and 3-year FU ESTOIH_ITT and PP analysis

Analysis	Short-stitch group	Long-stitch group	OR; 95% CI	*p* value
1-year ITT	3.3%, (7/210)	6.4%, (13/204)	1.95, 95% CI [0.77–5.05]	0.173
1-year PP	4.2%, (7/165)	8.2%, (13/158)	2.02, 95% CI [0.79–5.21]	0.168
3-year ITT	7.58%, (15/198)	10.45%, (21/201)	1.42, 95% CI [0.71–2.84]	0.316
3-year PP	10.79%, (15/139)	15.67%, (21/134)	1.53, 95% CI [0.75–3.12]	0.233

### Outcomes

#### Incisional hernias

**Fig. 2:**
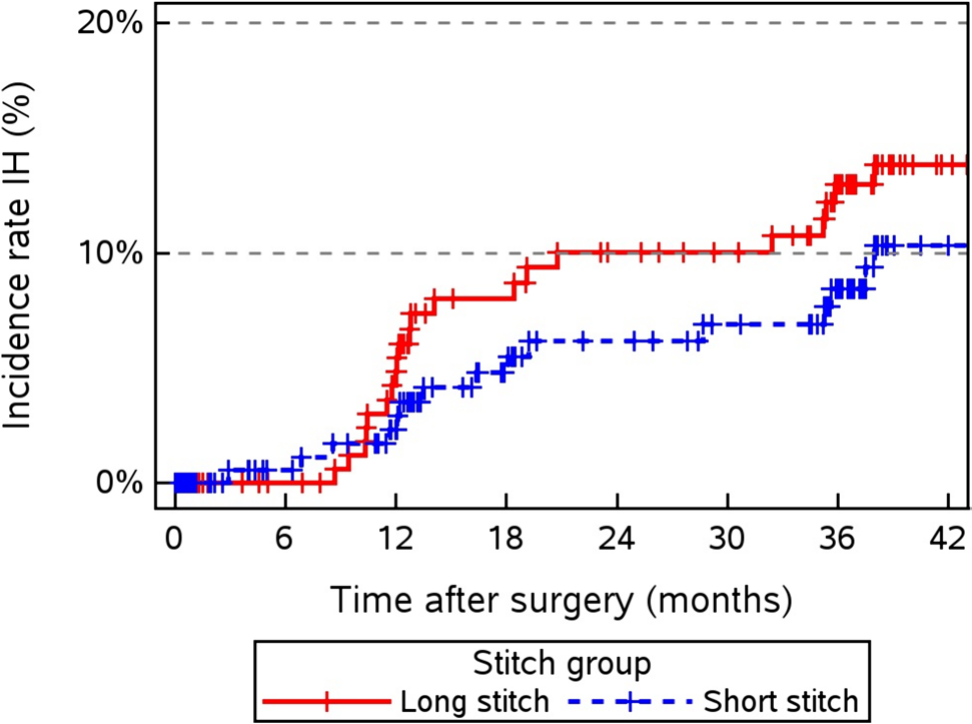
Incisional hernia incidence

The primary outcome of incisional hernia after 1 year has been previously published [9], and the data are provided in Table 1. Here, we report the 3-year results. In the intention-to-treat analysis, the incisional hernia rate was 7.58% (15/198 patients) for the short-stitch technique compared to 10.45% (21/201 patients) for the long-stitch technique, which was not significant (*p* = 0.31, OR 1.4233, 95% CI [0.7112–2.8485]). The development of the hernia rate is shown in the time course after the 1st year, with a constant gap between the short and long-stitch techniques (Fig. 2). Compared with 1 year postoperatively, incisional hernias across groups increased from 4.83% (20/414 patients) to 9.02% (36/399 patients, *p* = 0.0183).

The per-protocol analysis showed a 10.79% (15/139 patients) hernia rate in the short-stitch group vs. 15.67% (21/134) in the long-stitch group (*p* = 0.233, OR 1.5363, 95% CI [0.7554–3.1246], Table 1). For both groups combined, the hernia rate increased from 6.19% (20/323 patients) 1 year postoperatively to 13.19% (36/273 patients) in the per-protocol analysis.

Most incisional hernias were found in the epigastric region (55%) and 22% in the umbilical region, with no significant difference between the treatment groups (Table 2). Half of the incisional hernias were < 4 cm in size, 11 hernias (30%) showed a range 4–10 cm and a minority of hernias were > 10 cm (Table 2). The distribution of hernia sizes was comparable between the two treatment groups. In total, 36% (13/36) of the hernias were surgically treated, most often using the sublay technique. The number of operated hernias was higher in the short-stitch group than in the long-stitch group (7/15 patients (46%) vs. 6/21 patients (29%), Table 2).

**Table 2:** Characteristics of incisional hernias 3-year FU

	Short-stitch group (*n* = 15)	Long-stitch group (*n* = 21)	Total (*n* = 36)
EHS classification localization			
Epigastric	9	11	20
Umbilical	3	5	8
Infraumbilical	1	1	2
Subxiphoidal	1	2	3
Suprapubic	0	0	0
Missing	1	2	3
EHS classification size (cm)			
< 4 cm	7	11	18
≥ 4–10 cm	4	7	11
> 10 cm	1	1	2
Missing	3	2	5
Need for surgical repair			
Yes	7	6	13
No	8	15	23

### Quality of life

**Fig. 3:**
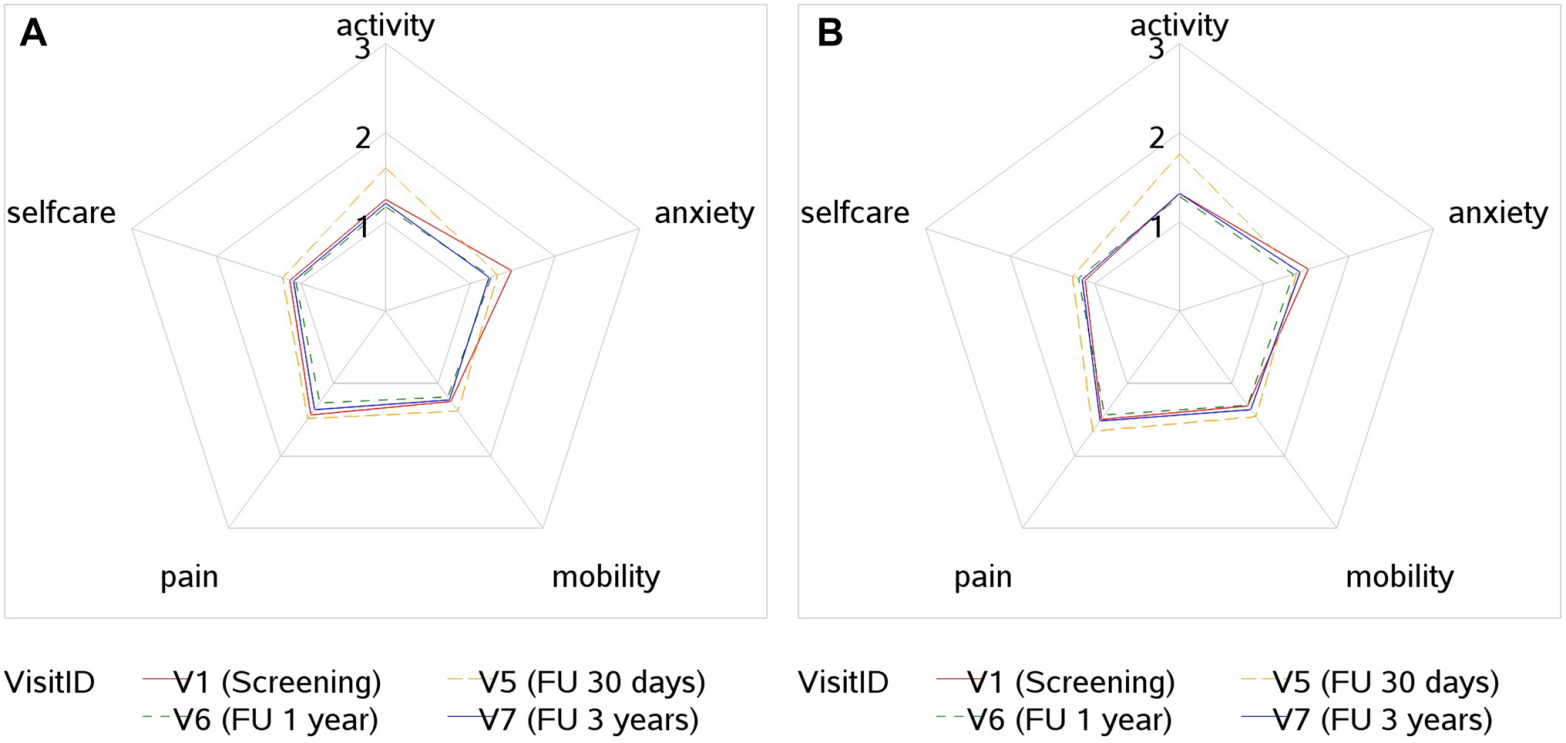
**A** QoL short-stitch group 3 years postop, **B** QoL long-stitch group 3 years postop

Quality of life from screening to 3 years postoperatively is shown in Table 3. At the 3-year follow-up visit, valid EQ-5D questionnaires were completed by 224 of 273 patients (82%). The quality of life was higher in the short-stitch group than in the long-stitch group 1 year postoperatively, and a significant increase was found from screening to 1 year after surgery. No further improvement was found at 3 years postoperatively in either group. The VAS values at 3 years were congruent with the values found at the 1-year follow-up in both treatment groups; there was no secondary deterioration in the quality of life. The advantages of the short-stitch suture technique in the EQ-5D scale, EQ-5D index, and the EQ dimensions of self-care and pain 12 months postoperatively were no longer present at the 3-year follow-up (Fig. 3A, B)

**Table 3:** QoL EQ VAS until 3 years postop

EQ scale (0–100)	*N*	Min	Q1	Median	Q3	Max	Mean	StdDev
All								
All-stitch group	1192	0.00	65.00	80.00	90.00	100.00	74.82	18.38
Short stitch	617	0.00	65.00	80.00	90.00	100.00	76.23	18.24
Long stitch	575	10.00	60.00	75.00	90.00	100.00	73.32	18.42
VisitID V1 (screening)								
All-stitch group	367	0.00	65.00	80.00	90.00	100.00	75.26	18. Dez
Short stitch	187	0.00	65.00	80.00	90.00	100.00	75.90	18.19
Long stitch	180	10.00	60.00	80.00	90.00	100.00	74.59	18. Aug
V5 (FU 30 days)								
All -stitch group	314	10.00	60.00	75.00	85.00	100.00	70.30	18.17
Short stitch	162	10.00	58.00	75.00	85.00	100.00	70.31	17.88
Long stitch	152	10.00	60.00	70.00	85.00	100.00	70.28	18.54
V6 (FU 1 year)								
All-stitch group	288	10.00	70.00	80.00	90.00	100.00	77.59	17.37
Short stitch	152	10.00	70.00	81.00	92.50	100.00	80.44	16.70
Long stitch	136	20.00	65.00	80.00	90.00	100.00	74.41	17.61
V7 (FU 3 years)								
All-stitch group	223	10.00	65.00	80.00	90.00	100.00	76.91	19.27
Short stitch	116	20.00	70.00	82.50	95.00	100.00	79.50	18.69
Long stitch	107	10.00	60.00	80.00	90.00	100.00	74.09	19.58

## Discussion

In this follow-up analysis of the ESTOIH trial, we report an increase in incisional hernia rates between one and 3 years postoperatively, with no significant difference between treatment groups. To our knowledge, three prospective trials investigating the short-stitch technique have been performed, and this is the first report of 3-year hernia rates from such a trial.

It has been shown that a significant number of incisional hernias occur after the first postoperative year. In an observational study by Itatsu et al., incisional hernia rates doubled from 5.2% at 12 months to 10.3% at 24 months [13], and in a follow-up analysis of the INSECT and ISSAAC trials, incisional hernias increased from 12.6% at 1 year to 22·4% 3 years after surgery [5]. These findings are confirmed in the per-protocol analysis of the ESTOIH trial, which hernia rates increased from 6.2% (20/323 patients) after 1 year to 13.2% (36/273 patients) after 1 years.

In the recently published 5-year follow-up analysis of the PRIMA trial [14], the authors reported an increase in incisional hernias from 30% at 2 years to 53.4% for the long-stitch technique without mesh reinforcement in high-risk patients (BMI ≥ 27 kg/m^2^ or abdominal aortic aneurysm). This represents a remarkable 78% increase, indicating the necessity for extended follow-up in trials with incisional hernia as the primary outcome. Anticipating a similar increase, a follow-up analysis 5 years postoperatively was included in the ESTOIH trial protocol [7].

The non-significant difference in hernia development between the treatment groups found 1 year after laparotomy closure [9] remained discernible after 3 years. However, the higher number of events did not result in statistical significance, and it remains to be seen whether a more evident advantage can be demonstrated with a prolonged follow-up.

The per-protocol analysis included only patients treated according to their assigned treatment group who completed 3-year follow-up or experienced an event before that time. Therefore, it yields a higher hernia rate than intention-to-treat analysis, which includes all randomized patients. In this context, the 13.2% hernia rate at 3 years across the treatment groups is comparable to previous findings [15]. Given that the majority of earlier studies used poly-dioxanone-based sutures, the low hernia rates in the ESTOIH trial may be attributed in part to the distinctive properties of the poly-4-hydroxybutyrate-based sutures applied in both treatment arms.

First, poly-4-hydroxybutyrate is characterized by 90% elasticity compared to 50% for poly-dioxanone [16], which reduces tension on the fascia. Improved tissue perfusion may lead to low rates of surgical site infections, better mature collagen formation, and ultimately, fewer early hernias. Second, the 50% basic strength retention time (100 vs. 42 days) and complete resorption time (390 vs 180–210 days) were significantly longer for poly-4-hydroxybutyrate compared for poly-dioxanone [7]. The delayed resorbability of the suture material is thought to support scar formation and wound healing over time, leading to fewer delayed hernias. Since incisional hernias developed early and late during follow-up, none of these mechanisms appear to be more important than the others. Future randomized trials should be designed to compare the different suture materials.

However, the hernia rate in the ESTOIH trial was also lower than that in the ISSAAC trial, which used poly-4-hydroxybutyrate-based sutures, suggesting that the suture material alone cannot explain the effect. In this regard, a very high degree of suture technique standardization in both treatment arms may have played a role. The study protocol included a detailed section on the preparation of the fascia, stitch length, suture width and their ratio, and knot tying. Additionally, surgeons were trained by the principal investigator on-site, and a video explaining the suture technique was distributed to surgeons participating in the study at a later time. As a result, high SL:WL ratios were achieved in both treatment arms, as recommended by Israelsson et al. [17, 18]. Standardization of the suture technique has been shown to reduce early suture complications [19], and it is possible that in the ESTOIH trial, we are witnessing its positive long-term effects. The effect of a standardized suture technique and training may be perceived as a beneficial form of bias.

The ESTOIH trial has several strengths, including its prospective multicenter parallel-group double-blind randomized-controlled design, which enhances the reliability and validity of the findings. The prolonged follow-up is another strong point. However, there are weaknesses, such as the unexpectedly low incidence of incisional hernias, which may have limited the ability to detect statistically significant differences between groups. Additionally, the high number of surgeons (over 100) involved could have introduced variability in surgical technique, potentially affecting the outcomes. Slow recruitment, changes to inclusion criteria during the trial and patient dropouts might also affect the generalizability of the results.

## Conclusion

The ESTOIH trial’s 3-year follow-up demonstrated a significant increase in incisional hernia rates from 1 to 3 years postoperatively, while affirming the long-term safety of the short-stitch technique with a poly-4-hydroxybutyrate-based suture. However, no significant advantage over the standard long-stitch technique was identified.

## Data Availability

Individual de-identified participant data will be made available beginning 6 months after publication and ending after 5 years. The data will be shared with investigators who provide a methodologically sound proposal to the sponsor. The proposals are directed to petra.baumann@aesculap.de. Data requests need to sign a data access agreement. Data were available for 5 years on a third-party website. The trial protocol has been published with open access to the journal Trials. Fortelny RH et al. Effect of suture technique on the occurrence of incisional hernia after elective midline abdominal wall closure: study protocol for a randomized-controlled trial. Trials 2015; 16(1):52.
